# Effects of Phenylethanoid Glycosides Extracted from *Herba Cistanches* on the Learning and Memory of the APP/PSI Transgenic Mice with Alzheimer's Disease

**DOI:** 10.1155/2021/1291549

**Published:** 2021-01-18

**Authors:** Jianhua Yang, Bowei Ju, Junping Hu

**Affiliations:** ^1^Department of Pharmacy, The First Affiliated Hospital, Xinjiang Medical University, Urumqi 830011, China; ^2^College of Pharmacy, Xinjiang Medical University, Urumqi 830011, China; ^3^Department of Pharmacy, The Fifth Affiliated Hospital, Xinjiang Medical University, Urumqi 830011, China

## Abstract

**Background:**

To investigate the effects of phenylethanoid glycosides (PhGs) extracted from *Herba Cistanches* on the behavioral and cognition capacity of the APP/PSI transgenic mice with Alzheimer's disease (AD).

**Methods:**

AD mice were randomly divided into the control group, model group, donepezil group, PhG groups, and verbascose group, respectively. Three weeks later, the animals were subject to behavioral and cognition evaluation by the nesting test, Morris water maze test, and step-down test.

**Results:**

The cognition capacity in these groups showed a significant increase compared with that in the model group. The step-down test indicated that the errors induced by the memory decrease in the PhG groups and verbascose group showed a significant decrease compared with those in the model group (*P* < 0.05).

**Conclusions:**

PhGs attenuated the cognitive dysfunction features of the APP/PSI transgenic gene. Besides, PhGs were the active components for the anti-AD activity of *H*. *Cistanches*.

## 1. Background

Alzheimer's disease (AD) is an age-related degenerative disease of the central nervous system (CNS) featured by progressive cognitive dysfunction and memory impairment [1–3]. With the aging process and extension of life span worldwide, the prevalence of AD is on an increasing trend, which results in high morbidity, long disease course, and high treatment expenditure. To date, the mechanism of AD is still not well defined [4–7]. Therefore, it is urgent to investigate the mechanism of AD and develop an effective method for the prevention, control, and treatment of AD accordingly.

To date, there is still no effective method for the prevention and treatment of AD, and most of the agents used in clinical settings are used for symptomatic treatment. The approved agents for AD by FDA include cholinesterase inhibitors (e.g., donepezil) and excitatory amino acid receptors (e.g., memantine) that are considered to attenuate the symptoms and improve the cognitive/behavioral capacities. Nevertheless, efficiency is not satisfactory due to limited treatment responses after administration [8–10]. Traditional Chinese medicine, with a long history, based on thousands of years of experience, shows extensive sources and several advantages compared to the chemical agents, including multiple targets, excellent safety, few side events, and suitability for long-term administration. These herbal medicines present superiority to other agents in terms of treating chronic and refractory diseases. Additionally, great potency was reported in these agents in the treatment of AD [11–13]. Therefore, increasing efforts have been made on the development of new agents for AD from herbal medicines based on modern techniques.

*Herba Cistanches*, known as Rou Cong-Rong in Chinese, are an endangered wild species that are mainly distributed in the arid lands and warm deserts of northwestern China. *Herba Cistanche* is applied as a tonic and/or in a formula for chronic renal disease, impotence, and senile constipation with a history of two thousand years. The chemical constituents of *Herba Cistanche* mainly consist of volatile oils, nonvolatile phenylethanoid glycosides (PhGs), iridoids, lignans, alditols, oligosaccharides, and polysaccharides. There have been an increasing number of studies focusing on antiaging, especially the animal models established using A*β*25-35, D-galactose, Al_2_O_3_, quinolinic acid, and scopolamine. These studies indicated that the PhGs could contribute to memory, attenuation of oxidative stress in brain tissues, inhibition of nerve cell apoptosis, and decline of acetylcholine esterase [14–16].

In the ancient prescriptions, *H*. *Cistanche* is frequently for nurturing the kidney and inhibiting the aging process. In modern times, the herb has been approved to be effective for treating certain diseases with satisfactory safety. Besides, it has been utilized in individuals living in Mainland China [17]. In 2016, the CFSA approved the safety of *H*. *Cistanche* in food sources, and then, in 2018, the National Health Commission of the PRC issued that it could serve as a food source and a candidate for the research and development of new drugs [18, 19]. To our best knowledge, the active components of *H*. *Cistanche* are PhGs, among which echinacoside and verbascose serving as the most representative components have been listed as a quality index for *H*. *Cistanche* by the *Chinese Pharmacopoeia*. For instance, the PhGs of *H*. *Cistanche* may serve as a food additive after purification.

Our previous studies indicated that the PhG extracted from the *H*. *Cistanche* was far more than 80% [11]. Besides, the PhG monomer and the verbascose were high in content and served as the major active components. Extensive studies indicated that the total PhGs and PhG monomer (e.g., verbascose and echinacoside) presented significant anti-AD efficiency. For example, animal studies showed that total PhGs, verbascose, and echinacoside could significantly contribute to the increase of the learning/memorizing capacity and cognitive behaviors, downregulate the oxidative stress in brain tissues, inhibit the apoptosis of nerve cells, and decrease the generation of A*β* in brains and the acetylcholine esterase activity [20–25]. In vitro experiments revealed that PhGs could act on the AD cellular model, bring down the oxidative stress, remove the free radical, inhibit the amyloid-*β* aggregation, and decrease the neuron loss and nerve cell apoptosis [20, 25, 26]. Furthermore, PhGs could attenuate the cognitive dysfunction and the generation of A*β*1-40 and A*β*1-42 in the hippocampus in the APPswe/PSIdE9 transgenic mice. It can regulate the upstream and downstream targets of the insulin signaling pathway, which then improved the signal transmission of the insulin and regulated the brain energy metabolism, as well as the subsequent anti-AD capacity [27].

Animal models are crucial for disease-related research studies and pharmacy development. The AD model based on transgenic animals has been considered one of the most important advances as the transgenic AD animal model could mimic AD at a molecular level, which presented typical symptoms and signs. Besides, this model is superior to the other AD-related models in stability and reliability. Up to now, no transgenic AD model has been conducted to investigate the effects of the PhG monomer on the pathogenesis of AD. In this study, the APP/PSI AD mice model was established, followed by the determination of anti-AD activities of a PhG monomer termed as verbascose by evaluating the nesting test, Morris water maze test, and step-down test.

## 2. Methods

### 2.1. Agent

**Figure 1:**
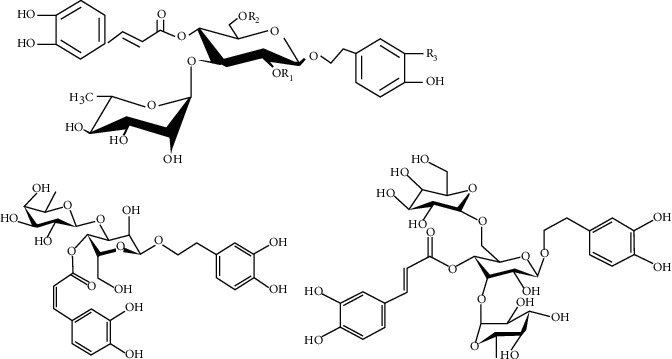
Graphic formula for PhGs, echinacoside, and verbascose.

The PhGs of *H*. *Cistanche* were prepared according to our conventional description [20]. The quality determined using the UV method was up to 87.6%. The echinacoside and verbascose content determined by HPLC was 37.8% and 17.7%, respectively. The graphic formula for PhGs, verbascose, and echinacoside is shown in Figure 1. Stems of *C*. *tubulosa* (Schrenk) Wight were collected in October 2016 from Xinjiang, China. The plant was identified by Dr. Junping Hu. All these voucher specimens (No. 201610) have been deposited at the Plant Herbarium, School of Pharmacy, Xinjiang Medical University, Xinjiang, China.

### 2.2. Animals

Ten male APP/PSI transgenic mice (3 months) and twenty female mice (male: 10; female: 10, 20-30 g) were purchased from the Model Animal Research Center of Nanjing University (SCXK 2015-0001, Nanjing, China). The animals were fed in the specific pathogen-free (SPF) chambers in the Animal Center of Xinjiang Medical University (SCXK 2011-0001, Xinjiang, China) on a SPF-grade diet. All the animals have free access to food and sterilized water. The study protocols were approved by the Ethics Committee of Xinjiang Medical University.

### 2.3. Proliferation and Identification of APP/PSI Transgenic Mice

The newborn transgenic mice (2-3 weeks) were subject to tail cutting with a length of 0.5 cm for DNA extraction. The tail was placed in an Eppendorf tube, followed by adding prolease K until a concentration of 400 *μ*g/ml. Subsequently, the mixture was vortexed at 55°C overnight and then was centrifuged at 13,000 rpm for 10 min at 4°C to obtain the supernatant. DNA extraction was performed using a commercial kit according to the manufacturer's instructions (Qiagen, Germany).

For the DNA amplification, specific primers for APP and PSI were designed as mentioned in Table 1. The amplification was conducted in a total volume of 20 *μ*l using the following conditions: 94°C predenaturation for 5 min, followed by 35 cycles of denaturation at 94°C for 30 sec, 65°C for 35 sec, and 72°C for 45 sec, as well as 72°C for 3 min. The produced DNA products were subject to agarose gel electrophoresis at a constant voltage of 100 V for 30 min. Finally, the images were visualized using a ChemiDoc MP system.

### 2.4. Grouping

Using the randomization generated by SPSS 18.0 software, the AD mice were randomly divided into the (i) control group (*n* = 10, C57BL/6J mice); (ii) model group (*n* = 10); (iii) donepezil group (*n* = 10, 0.65 mg/kg, intragastric administration); (iv) PhG groups subject to PhG via lavage with a concentration of 250 mg/kg (*n* = 10), 125 mg/kg (*n* = 10), and 62.5 mg/kg (*n* = 10); and (v) verbascose group (*n* = 10, 125 mg/kg, intragastric administration). Animals used in groups (ii)-(v) were APPswe/PSIdE9 transgenic mice. The lavage was performed at the age of 6 months and lasted for 3 months with a frequency of once per day. Animals in the control and model groups were subject to injection of double distilled water intragastric administration.

### 2.5. Nesting Test

The animals were fed separately for at least 24 h before the test in a standard cage (28 × 12 × 16 cm), filled with sterilized wood shavings with a thickness of 1 cm. Initially, the latency time was monitored at the beginning of the test, and a total time of 120 was obtained. The nesting score was calculated as previously described [28].

### 2.6. Morris Water Maze Test

The apparatus consisted of a circular pool filled with water (23 ± 2°C and 40 cm in depth) located in a test room with white walls and several cues on them. A platform (10 cm in diameter) was immersed 1 cm under the surface of the water in one of the four identical quadrants. The location of the platform was fixed throughout the acquisition trials, during which mice were individually placed in the water facing the wall of the pool in order to negate any directional influence. On days 1 and 2, the acquisition trial test was conducted including 4 sessions per day for 6 consecutive days. For each session, the mice were allowed to escape by swimming to the invisible platform and the escape latency (i.e., the time required to locate and climb onto the platform) and swimming speed were recorded with the cutoff time 60 s. Mice that fail to find the platform within 60 s were guided to the platform manually and allowed to stay on it for 10 s. On day 11, the acquisition trial test was performed again for the last 6 consecutive days after all the drug treatments.

### 2.7. Step-Down Test

About 24 h after the last drug medication, the animals were required to be in the step-down facility and adaptive environment for 3 min. The time was set for 5 min, and the voltage was 32 V. Upon stimulation, the mice tried to jump onto the platform and then were timed in the presence of infrared detector signal changes. The latency time was recorded as the time from the platform to the slot bottom for the first time. In cases of not jumping off the platform, the latency time was recorded as 5 min. Then, the total stimulation frequency was recorded after the latency stage. The test was accomplished in 2 days. Learning and memory were observed on day 1 and day 2, respectively. The latency stage and error frequency were recorded for each mouse.

### 2.8. Statistical Analysis

SPSS 18.0 software was used for the statistical analysis. Data were presented as mean ± standard deviation. Student's *t*-test was performed for the intergroup comparison. *P* < 0.05 was considered to be statistically significant.

## 3. Results

### 3.1. Genotype Identification

**Figure 2:**
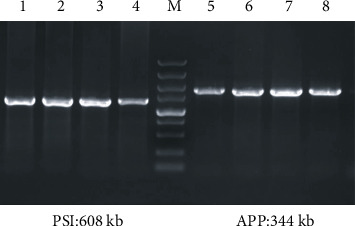
Gel analysis of APP/PSI transgenic mice using PCR amplification. Lanes 1-4: APP/PSI-positive mice (PSI gene). Lanes 5-8: APP/PSI-positive mice (APP gene).

Given the fact that genome DNA served as the template for amplification, two bands with a length of 344 kb and 608 kb were obtained in the F1 generation, respectively. This was consistent with the length of the *APP* gene and *PSI* gene (Figure 2).

### 3.2. Nesting Score

**Figure 3:**
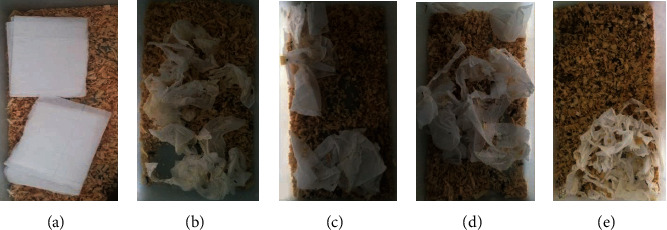
Evaluation standard for the nesting behavior of the mice. (a) Score 0. (b) Score 1. (c) Score 2. (d) Score 3. (e) Score 4.

**Figure 4:**
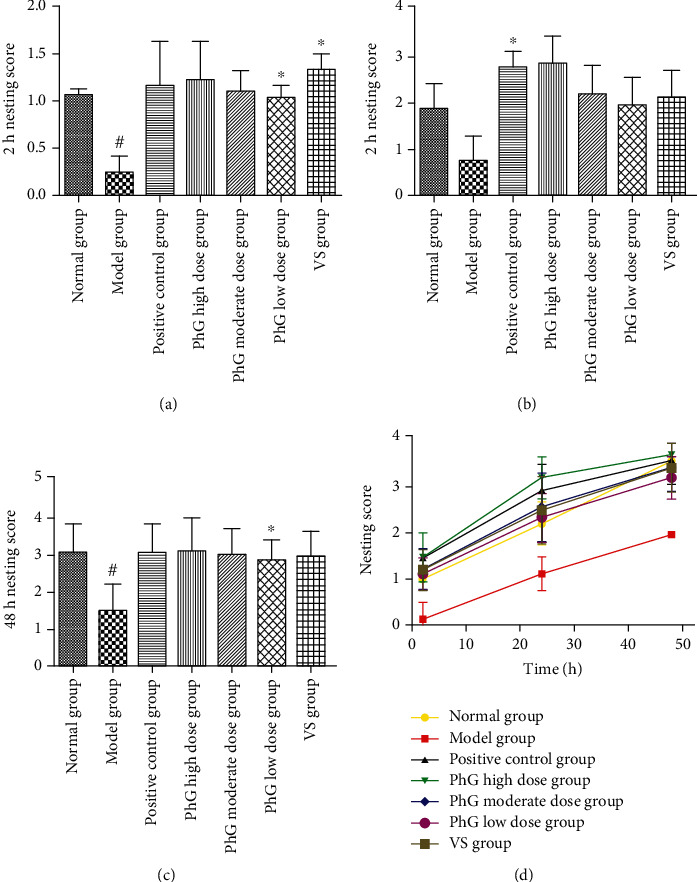
Nesting score in each group at 2 h (a), 24 h (b), and 48 h (c). Changes in the score in each group (d).

Five grades were divided into the nesting score (Figure 3). In the control group, nesting behavior was observed within a few minutes after placing the papers into the cage. In the model group, the exploration of the mice was comparatively decreased, and some animals may even present anxiety-related behaviors such as grooming and face washing, which triggered a delay in nesting behavior. Most of these animals showed a score of 1 or 2. Nevertheless, a significant increase was noticed in the nesting score, which presented in a time-dependent manner after PhG treatment (Figure 4).

### 3.3. Effects of PhGs on Navigating Transgenic AD Mice in the Latency Stage

With the extension of drill time, the latency time in each group showed a significant decrease. The first 2 days served as drill time. On day 3, the latency time in the model group showed significant extension compared with that in the normal control (*P* < 0.01). Similarly, on days 4 and 5, the latency time in the model group showed significant extension compared with that in the normal control (*P* < 0.05). On day 3, the latency time in the donepezil group showed a significant decrease compared with that in the model group (*P* < 0.05). The latency time in the PhG groups showed a significant decrease in a dose-dependent manner compared with that in the model group (*P* < 0.05, Table 2).

### 3.4. PhGs Significantly Attenuated the Swimming Trace of Transgenic AD Mice

**Figure 5:**
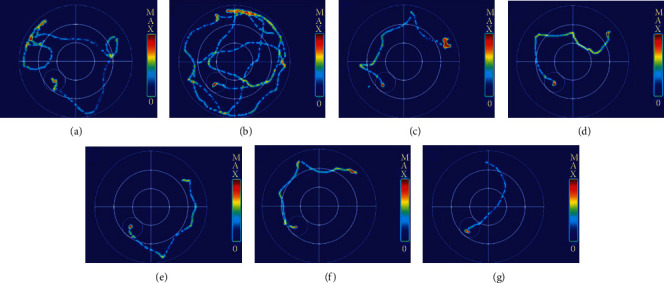
Morris water maze test traces. (a) Control. (b) Model group. (c) Positive control group. (d) PhG high-dose group. (e) PhG moderate-dose group. (f) PhG low-dose group. (g) Verbascose group.

In the model group, the swimming trace of the mice was in a typical manner of marginal searching. In the PhG groups and verbascose group, the swimming trace was in a linear type or tendency type. This indicated that PhG could significantly improve the special memory of APP/PSI transgenic AD mice (Figure 5).

### 3.5. Effects of PhGs on Special Exploration of AD Mice

The platform was retrieved on day 6 of the water maze test to investigate the special exploration capacity. The frequency of passing through the platform was recorded within 60 sec. Our data showed that a significant decrease was noticed in the frequency of passing through the platform in the model group, compared with the normal control (*P* < 0.01). Compared with the model group, a significant increase was noticed in the frequency of passing through the platform in the PhG groups and verbascose group (*P* < 0.05, Table 3).

As the platform was placed on the third quadrant, it was termed as the objective quadrant. Longer retention in the quadrant implied significant improvement in the learning and memory of the mice after the administration of agents. Our data showed that there was a significant decrease in the retention in the objective quadrant in the model group compared with the normal control (*P* < 0.01). Compared with the model group, a significant increase was noticed in the retention in the PhG groups, donepezil group, and verbascose group, respectively (*P* < 0.05, Table 4).

### 3.6. Effects of PhGs on the Step-Down Test in Transgenic AD Mice

The latency time in the model group was significantly lower than that in the control group (*P* < 0.01). In addition, donepezil could induce a significant increase in the latency time (*P* < 0.01). Similarly, the PhG groups and verbascose group also showed significantly longer latency time (*P* < 0.01). Moreover, the PhG groups and verbascose group showed a significant decrease in the error frequency in the step-down test induced by memory and learning impairment (*P* < 0.01). These indicated that PhGs contributed to the improvement of memory and learning capacity of transgenic AD mice (Table 5).

## 4. Discussion

AD is a degenerative nervous system disease that frequently occurs in the aged population. Unfortunately, the exact mechanism is still not well defined, which limits drug screening to some extent. To date, extensive studies have been conducted on drug development in view of animal research, and several AD animal models are available including models based on A*β*25-35, D-galactose, Al_2_O_3_, quinolinic acid, and scopolamine. These models trigger cognitive dysfunction, but they present disadvantages including the late onset of disease, large individual differences, and longer experimental duration that hampered mimicking the progressive and degenerative lesions [29–31]. With the advances of the transgenic technique in establishing AD animal models [27, 32, 33], great strides have been made in understanding the pathogenesis of AD together with the development of new drugs. Nevertheless, the pathogenesis of AD is rather complex, and only a few gene targets responsible for the onset of AD have been identified [34–36]. On this basis, the transgenic AD model deserves further application in drug development. Identification of gene sites associated with AD based on the transgenic AD model using the crossbreeding technique will contribute to the treatment of AD in clinical settings and establishing new AD models involving multiple virulence genes.

In this study, the selected APPswe/PSIdE9 model contributed significantly to APP metabolism, development, and pathogenesis of AD, as well as disease progression. For the proliferation and identification of transgenic mice, we extracted DNA from the blood samples and tissues, respectively. For the blood sample collection, in order to ensure the survival of animals, the sample was collected from the tail top, which was difficult to perform with less sample volume. Besides, it may induce injury to the animals and affect the subsequent analysis. In contrast, DNA extraction from tissues was usually performed at weeks 6-8, which was easy to perform and caused less injury to the growth of animals. Furthermore, there were no statistical differences in the DNA content and purity between these two methods. Therefore, DNA was extracted from the tissues for the identification of the genome of transgenic mice.

The typical clinical manifestations for AD are a progressive decrease in learning and memory. Thus, observation of learning and memory is crucial for the evaluation of drug efficiency. In this study, the nesting test was utilized together with the Morris water maze test and step-down test to investigate the effects of PhGs on the memory and learning of APP/PSI transgenic mice. In the navigation test, the latency time in the model group indicated a significant increase compared with that in the normal control (*P* < 0.05). Meanwhile, a significant decrease was noticed in the latency time in the donepezil group compared with the model group (*P* < 0.05). Compared with the model group, the latency time in the PhG groups showed a significant decrease in a dose-dependent manner (*P* < 0.05). In the special exploration, a significant decrease was noticed in the frequency of passing through the platform in the model group compared with the normal control (*P* < 0.01). Compared with the model group, the frequency of passing through the platform in the PhG groups and donepezil group showed a significant increase (*P* < 0.05). In the step-down test, the frequency of error in jumping induced by memory and learning impairment was significantly decreased after administration of PhGs (*P* < 0.01). The behavioral test revealed that PhG interference could significantly improve the memory and learning of the APP/PSI transgenic AD mice.

Traditional herbal medicine, with a long history, has been commonly utilized in treating chronic disease and refractory disorders. Currently, the development of new drugs on AD based on herbal medicine has been highly focused on the extraction of active components and/or compound preparation. Compared with the active extraction component, the single monomer component shows various advantages such as the utilization of low concentration, easy investigation of target sites, and better efficiency direction. Verbascose is a representative monomer of PhGs as it shows high content and activity. In this study, the verbascose group showed better anti-AD capacity compared with the PhG groups. This supports the research and development of anti-AD agents based on verbascose. The *H*. *Cistanches* show a long history in food utilization, dating back to the Han dynasty. Modern techniques approve that *H*. *Cistanches* are safe as food. In this study, we approved the potency of *H*. *Cistanches* in the fields of functional food, drug development, and health care, especially in the aged population and the fatigue individuals.

The animal model is crucial for research studies on disease and drug development. Recently, the transgenic technique has been used to establish the AD model, which can mimic the pathogenesis of AD at molecular levels, together with the typical symptoms and vital signs. These models have been proved to be effective in stability and reliability compared with the other AD models. In this study, the APP/PSI transgenic mice model that is well acknowledged for mimicking the pathological processes of AD is utilized, which presented features of behavioral dysfunction, cognitive disorder, senile plaques, neurofibrillary tangles, and neuronic death [37–39]. In this study, we firstly utilized transgenic AD mice to investigate the effects of PhGs on learning and memory, which can provide helpful information for the research and development of anti-AD agents based on PhGs.

In summary, PhGs could attenuate the cognitive dysfunction features of the APP/PSI transgenic gene. Our study confirmed that PhGs were the active components for the anti-AD activity of *H*. *Cistanches*. It may pave the way for the development of new drugs for the treatment of AD.

## Figures and Tables

**Table 1:** Specific primers for the PCR amplification.

Name	Sequence	Length
APP	Forward: 5′-GACTGACCACTCGACCAGGTTCTG-3′ Reverse: 5′-CTTGTAAGTTGGATTCTCATATCCG-3′	344 bp
PSI	Forward: 5′-AATAGAGAACGGCAGGAGCA-3′ Reverse: 5′-GCCATGAGGGCACTAATCAT-3′	608 bp

**Table 2:** The latency time in the APP/PSI mice with AD.

Group	Day 1	Day 2	Day 3	Day 4	Day 5
Control (*n* = 10)	60.00 ± 0.00	59.33 ± 1.15	30.6 ± 11.96	13.43 ± 3.76	10.53 ± 4.64
Model group (*n* = 10)	60.00 ± 0.00	54.77 ± 8.89	50.9 ± 13.25^#^	50.87 ± 14.87^##^	50.37 ± 15.91^##^
Donepezil group (*n* = 10)	57.23 ± 1.66	58.10 ± 3.12	36.23 ± 9.58^∗^	42.17 ± 17.85	46.57 ± 11.68
PhG high-dose group (*n* = 10)	45.47 ± 20.20	38.3 ± 19.19	35.6 ± 16.68	22.57 ± 16.74^∗^	18.23 ± 8.82^∗∗^
PhG moderate-dose group (*n* = 10)	53.43 ± 6.92	44.9 ± 13.55	39.5 ± 27.62	29.67 ± 26.46^∗^	25.90 ± 28.84^∗^
PhG low-dose group (*n* = 10)	60.00 ± 0.00	58.57 ± 2.48	45.7 ± 19.98	34.17 ± 24.72	21.40 ± 9.79^∗^
Verbascose group (*n* = 10)	55.21 ± 7.48	42.23 ± 15.20	35.85 ± 11.39	28.77 ± 19.47^∗^	23.66 ± 18.29^∗^

^##^ *P* < 0.01 vs. the control; ^#^*P* < 0.05 vs. the control; ^∗∗^*P* < 0.01 vs. the model group; ^∗^*P* < 0.05 vs. the model group.

**Table 3:** Frequency of passing through the platform in the APP/PSI mice with AD.

Group	Frequency
Control (*n* = 10)	3.20 ± 0.45
Model group (*n* = 10)	1.75 ± 0.50^##^
Donepezil group (*n* = 10)	3.25 ± 0.89^∗∗^
PhG high-dose group (*n* = 10)	4.44 ± 1.01^∗∗^
PhG moderate-dose group (*n* = 10)	3.56 ± 0.53^∗∗^
PhG low-dose group (*n* = 10)	2.75 ± 0.71^∗^
Verbascose group (*n* = 10)	3.26 ± 0.88^∗∗^

^##^ *P* < 0.01 vs. the control; ^#^*P* < 0.05 vs. the control; ^∗∗^*P* < 0.01 vs. the model group; ^∗^*P* < 0.05 vs. the model group.

**Table 4:** Retention time in the APP/PSI mice.

Group	Retention time (s)
Control (*n* = 10)	17.12 ± 0.76
Model group (*n* = 10)	8.40 ± 0.80^##^
Donepezil group (*n* = 10)	16.29 ± 1.26^∗∗^
PhG high-dose group (*n* = 10)	17.62 ± 1.05^∗∗^
PhG moderate-dose group (*n* = 10)	16.03 ± 0.77^∗∗^
PhG low-dose group (*n* = 10)	15.01 ± 0.94^∗^
Verbascose group (*n* = 10)	15.62 ± 1.13^∗∗^

^##^ *P* < 0.01 vs. the control; ^#^*P* < 0.05 vs. the control; ^∗∗^*P* < 0.01 vs. the model group; ^∗^*P* < 0.05 vs. the model group.

**Table 5:** Step-down frequency in the APP/PSI mice with AD.

Group	Latency (s)	Error frequency
Control (*n* = 10)	144.73 ± 15.37	4.50 ± 1.05
Model group (*n* = 10)	67.87 ± 7.14^##^	5.67 ± 0.82^#^
Donepezil group (*n* = 10)	127.90 ± 4.72^∗∗^	3.83 ± 0.98^∗∗^
PhG high-dose group (*n* = 10)	96.05 ± 5.63^∗∗^	3.50 ± 1.05^∗∗^
PhG moderate-dose group (*n* = 10)	118.08 ± 5.26^∗∗^	2.83 ± 0.75^∗∗^
PhG low-dose group (*n* = 10)	135.48 ± 12.26^∗∗^	2.00 ± 0.63^∗∗^
Verbascose group (*n* = 10)	111.66 ± 4.88^∗∗^	2.56 ± 0.82^∗∗^

^##^ *P* < 0.01 vs. the control; ^#^*P* < 0.05 vs. the control; ^∗∗^*P* < 0.01 vs. the model group; ^∗^*P* < 0.05 vs. the model group.

## Data Availability

All the data were available upon appropriate request.

## Abbreviations

PhGs: Phenylethanoid glycosides
AD: Alzheimer's disease
CNS: Central nervous system.
